# A Simple Device for Measuring Sit-to-Stand Movements and Cardio-Metabolic Diseases: A Cross-Sectional Study in a Health Check-Up Population Over 20 Years Old

**DOI:** 10.7759/cureus.51918

**Published:** 2024-01-08

**Authors:** Takahiro Hokimoto, Jou-Yin Chen, Rupa Singh, Yukiko Wagatsuma

**Affiliations:** 1 Department of Clinical Trial and Clinical Epidemiology, University of Tsukuba, Tsukuba, JPN

**Keywords:** chronic diseases, lower limb muscle strength, diabetes mellitus type 2, sit-to-stand movement, ground reaction force, muscle strength, preventive medicine, cardiovascular disease

## Abstract

Introduction

Several studies have reported the usefulness of measuring lower limb muscle strength through the motion of standing up using a reaction force measuring device positioned on the ground. There is inadequate information on the association between cardiovascular disease risk factors and ground reaction force (GRF) during standing up. Therefore, this study estimated the association between GRF by sit-to-stand movements and cardiovascular disease risk factors in a health check-up population.

Methods

This cross-sectional study included 1,182 healthy participants without chronic diseases who underwent periodic health check-ups from August 2019 to December 2020. The study included individuals aged ≥20 years who underwent a standing test from an initial seated position in a chair. A sit-to-stand force analyzer was used to measure GRF, and health status information was collected at enrollment. The relationships between blood test data and each measurement obtained from GRF measurements (forth/body mass (F/M), rate of forth development/body mass (RFD/M), and stable time) were parsed according to sex using linear regression analysis coordinated by age. GRF measurements and their relationships with cardiovascular disease risk factors were assessed using logistic regression analysis, adjusted for age and sex.

Results

A total of 1,182 participants was included in this study, with male participants accounting for 61.5%. The study participants had a median age of 57.0 years (IQR: 47.0-63.0). After adjusting for age, F/M was positively associated with high-density lipoprotein cholesterol in male (β=22.59, p<0.001) and female participants (β=20.35, p=0.011) and negatively associated with plasma glucose in male (β=-16.25, p=0.008) and female participants (β=-18.78, p=0.028). Stable time (time required to be stabilize after standing up movement) was positively associated with hemoglobin A1c levels in male (β=0.55, p=0.001) and female participants (β=0.56, p=0.036). Logistic regression analysis adjusted by age and sex showed that a lower F/M ratio was associated with hypertension, hyperlipidemia, and diabetes mellitus (adjusted odds ratio (aOR) =1.60, p=0.01; aOR=1.75, p=0.001; and aOR=2.23, p=0.002, respectively). Lower RFD/M was associated with hyperlipidemia and diabetes mellitus (aOR=1.46, p=0.013 and aOR=1.63, p=0.045, respectively). A shorter stable time was associated with diabetes mellitus (aOR=0.39, p<0.001).

Conclusions

These findings suggest that lower limb function impairment, as assessed via standing-up movements using a GRF-measuring device, may relate to cardiovascular disease. Further research is needed to confirm this association.

## Introduction

Lower extremity muscle strength is paramount for maintaining a healthy life expectancy, as it is associated with decreased activities of daily living and falls. In recent years, the usefulness of measuring lower limb muscle strength while standing up using a ground reaction force (GRF) measuring device has been reported in several studies [1-4].

Standing is a common daily activity. A 30-second sit-to-stand test and a five-repetition-sit-to-stand test have been reported to measure the rising motion [5]. Both methods measure performance but cannot quantify muscle strength. Muscle strength measurement during standing up using GRF allows for uncomplicated measurement of muscle strength and power [6].

Previous studies have reported associations between muscle strength and various cardiovascular disease biomarkers, and grip strength was used as an evaluation method in healthy participants [7, 8]. However, its relationship with lower limb function remains to be investigated. In the older adult population, lower limb muscle weakness can lead to falls, which in turn leads to fractures and other mobility problems [9]. Muscle weakness has been reported not only in older adults but also in middle-aged and younger adults. Other factors have also been associated with muscle weakness [10]. Therefore, early measurement and intervention are necessary to prevent muscle weakness.

Various methods have been used to evaluate lower limb muscle strength, such as handheld dynamometers and Nottingham Power Hill [11, 12]. Recently, however, a method using an estimation formula from a field test of standing-up motion and a GRF measuring device has been reported to help evaluate lower limb muscle strength [6]. Standing up is a common activity that is easy to perform. The method using a GRF measuring device can effectively measure parameters such as muscle strength, muscle power, and balance and has been reported to be related to physical tests such as the timed up-and-go test [4].

However, most published reports that used GRF measuring devices have focused on healthy older adult participants. To the best of our knowledge, there are no comparable reports in healthy adults [1-4]. Hence, it is crucial to include a broader age range when presenting data on healthy adults. Consequently, our study seeks to estimate the relationship between GRF during sit-to-stand movements and cardiovascular disease risk factors within a population undergoing health check-ups.

## Materials and methods

Study area and population

**Figure 1:**
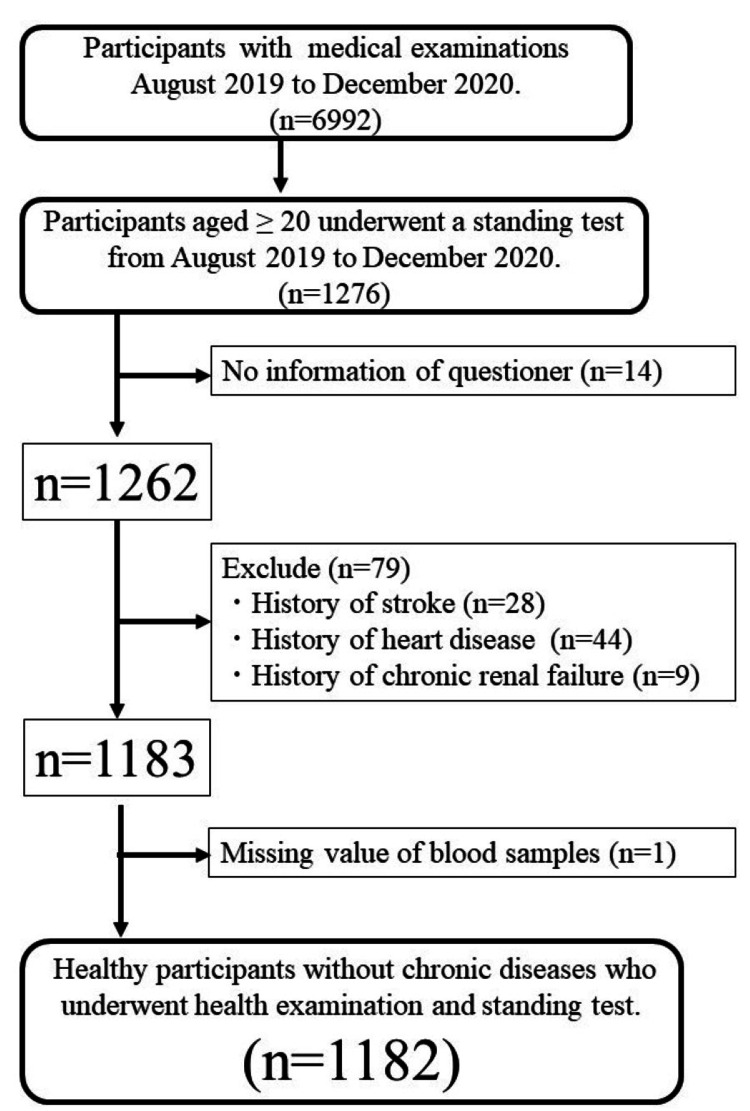
Study flow chart

The study participants were recruited from a health check-up population at a healthcare center located in a regional hospital (Mito Kyodo Hospital, Mito City, Japan). A total of 6,992 individuals underwent medical examinations at the healthcare center between August 2019 and December 2020. Of these, 1,276 aged ≥20 years underwent a standing test from an initial seated position in a chair. Fourteen of these patients could not receive our questionnaire, and an initial 79 were excluded because they had pre-existing conditions, such as stroke, heart disease, or chronic renal failure (28 individuals had strokes, 44 had heart disease, and nine had chronic renal failure). One individual was excluded due to missing data. Ultimately, 1,182 individuals were included in this study. Figure 1 illustrates the participant recruitment process.

Study design

This cross-sectional study included healthy adult participants without chronic diseases who underwent health check-ups and standing tests between August 2019 and December 2020. Inclusion criteria were individuals of both sexes who were aged ≥20 and who underwent health check-ups and standing tests. Exclusion criteria were a history of stroke, heart disease, or chronic renal failure. Demography, anthropometric variables, clinical values, and GRF measurements were collected during the specified study period.

Measurements

This study included participants who underwent a standing test. A previous study has reported the procedure and the methodology of this test [1, 2]. Vertical GRF was measured using a Zarits BM-220 sit-to-stand force analyzer (Tanita Co. Ltd., Japan). GRF (kgf) at the time of movement was recorded using a computer. Participants began by sitting on a chair at a height of 40 cm, and they crossed their arms in front of their chests. The participants then performed movements to stand up as soon as possible after receiving a signal from a gauger. Each participant performed the task three times according to the instructions delivered via video. The GRF was analyzed in terms of force/body mass (F/M) (maximum GRF/body mass), rate of force development (RFD)/M (rate of force development/body mass), and stable time (time between peak GRF and cessation of GRF fluctuation). RFD was divided by the time between the maximum and smallest GRF. We used the trial with the highest RFD/M value for the analysis.

Anthropometric measurements of height and weight were performed by medical staff using the Tanita DC250 Total Body Composition Analyzer (Tanita Co. Ltd., Japan). Height and body mass were measured in the standing position without shoes or heavy clothes. Body mass index was calculated as the body mass in kilograms divided by the height in meters squared [13]. Medical staff measured systolic blood pressure (SBP) and diastolic blood pressure (DBP) with the person in a sitting position. Blood pressure was measured up to twice. An average of the two measurements was considered the final value. In the case of one measurement, this one value was considered for analysis.

Blood samples were collected by medical staff after the individual's overnight fasting; samples were analyzed in the hospital's clinical laboratory. Blood analyses included high-density lipoprotein cholesterol (HDL-C), low-density lipoprotein cholesterol (LDL-C), triglyceride (TG), fasting plasma glucose (PG), and hemoglobin A1c (HbA1c) levels. We assessed the patients for a history of stroke, heart disease, chronic renal failure, and drug use using a self-report questionnaire of 22 items, a set of standardized questions for chronic disease control specified by the Ministry of Health and Welfare, Japan. The complete questionnaire is provided in Supplemental File S1. We used information on medical history and internal medication from the questionnaire.

Hypertension (HT) was defined as SBP ≥140 mm Hg, DBP ≥90 mm Hg, or current medication for HT [14]. Hyperlipidemia (HL) was defined as LDL-C ≥140 mg/dL, HDL-C <40 mg/dl, triglyceride ≥150 mg/dL, or current medication for HL [15]. Diabetes mellitus (DM) was defined as HbA1c ≥6.5 mmol/mol, fasting PG level of 126 mg/dL, current medication for DM, or taking insulin for DM [16].

Statistical analysis

Participants' GRF measurements (F/M, RFD/M, and Stable time), age continues, blood pressure, anthropometric measurements, and blood sample data are shown as the mean value and standard deviation or median and interquartile ranges for continuous variables. The total frequency and percentages were used for categorical variables.

GRF measurements were categorized according to sex and age (20-39, 40-49, 50-59, 60-69, and ≥70 years). A t-test was used to compare the sex, and a one-way analysis of variance was used to compare each age group by sex.

The relationships between blood data and each measurement obtained from GRF measurements were parsed and adjusted to sex using linear regression analysis adjusted by age. GRF measurements and their relationships with circulatory organ risk factors were assessed using logistic regression analysis, adjusted for age and sex. Participants were then divided into three groups based on tertile scores of the GRF measurements; these groups were utilized for logistic regression analysis. All statistical analyses were performed using IBM SPSS Statistics for Windows (version 25; IBM Inc., Armonk, NY, USA).

Ethical considerations

Written informed consent was obtained from all the participants. This study was approved by the Ethics Review Committee of the Faculty of Medicine at the University of Tsukuba (Approval No.: 1000) and adhered to the principles of the Declaration of Helsinki.

## Results

A total of 1182 participants participated in the physical examinations and GRF measurements. Table 1 presents the general characteristics of the participants. Of the total participants, 727 (61.5%) were males and 455 were females. The overall median interquartile range (IQR) age was 57.0 (47.0-63.0) years. HT, HL, and DM were found in 44.2%, 62.5%, and 11.1% of participants, respectively. The median (IQR) F/M was 1.46 (1.40-1.54) for the male participants and 1.34 (1.28-1.39) for the female participants. The median (IQR) RFD/M was 11.79 (10.47-13.08) for the male participants and 10.78 (9.78-11.81) for the female participants. The median (IQR) stable times were 1.04 (1.00-1.10) for the male and 1.01 (0.98-1.08) for the female. The GRF values for each sex are shown in Table 2. F/M and RFD/M values were lower for female participants than for male participants (F/M: p<0.001; RFD/M: p<0.001), and the stable time was longer for male participants than for female participants (p<0.001). The GRF values for each age group according to sex are shown in Table 3. F/M values decreased with age according to sex (p<0.001 for male participants; p<0.001 for female participants). RFD/M values decreased with age according to sex (p<0.001 for male participants; p=0.003 for female participants)

**Table 1:** General characteristics of participants Data were shown as medians (interquartile range) for continuous variables, number (percentage) for categorical variable. †Between peak GRF and cessation of GRF fluctuation HDL - figh-density lipoprotein; LDL - low-density lipoprotein; HbA1c - hemoglobinA1c; F/M - force/body mass; RFD/M - rate of force development/body mass

Characteristics	Total (n=1,182)	Male (n=727)	Female (n=455)
Age (years)	57.0 (47.0–63.0)	57.0 (46.0–63.0)	57.0 (48.0–64.0)
20–39 (%)	103 (8.7)	67 (9.2)	36 (7.9)
40–49 (%)	259 (21.9)	158 (21.7)	101 (22.2)
50–59 (%)	309 (26.1)	191 (26.3)	118 (25.9)
60–69 (%)	413 (34.9)	261 (35.9)	152 (33.4)
≥70 (%)	98 (8.3)	50 (6.9)	48 (10.5)
Height (cm)	165.8 (157.9–171.6)	170.0 (166.2–174.2)	156.7 (152.2–160.2)
Weight (kg)	64.1 (54.9–72.2)	69.2 (62.0–75.8)	53.4 (48.2–61.4)
Body mass index (kg/m^2^)	23.2 (21.0–25.7)	23.9 (21.9–26.0)	21.9 (19.9–24.8)
Systolic blood pressure (mmHg)	126.0 (116.0–135.0)	127.0 (116.0–136.0)	124.0 (113.0–134.5)
Diastolic blood pressure (mmHg)	81.0 (73.0–88.0)	82.0 (75.0–90.0)	78.0 (69.0–85.0)
HDL cholesterol (mg/dL)	59.0 (49.0–71.0)	54.0 (46.0–64.0)	66.0 (57.0–76.0)
LDL cholesterol (mg/dL)	121.0 (103.0–142.0)	121.0 (102.0–142.0)	122.0 (103.0–142.0)
Triglycerides (mg/dL)	92.0 (65.0–133.0)	101.0 (70.0–143.0)	80.0 (56.0–112.0)
Plasma glucose (mg/dL)	102.0 (96.0–110.0)	104.0 (97.0–113.0)	99.0 (93.0–106.0)
HbA1c (mmol/mol)	5.6 (5.4–5.9)	5.6 (5.4–5.9)	5.6 (5.4–5.9)
Hypertension (%)	475 (40.2)	331 (45.5)	144 (31.6)
Hyperlipidemia (%)	620 (62.5)	399 (54.9)	221 (48.6)
Diabetes mellitus (%)	131 (11.1)	99 (13.6)	32 (7.0)
F/M (kgf·kg^−1^)	1.41 (1.33–1.50)	1.46 (1.40–1.54)	1.34 (1.28–1.39)
RFD/M (kgf/s·kg^−1^)	11.35 (10.12–12.61)	11.79 (10.47–13.08)	10.78 (9.78–11.81)
Stable time (s)†	1.04 (0.99–1.09)	1.04 (1.00–1.10)	1.01 (0.98–1.08)

**Table 2:** Ground reaction force measurements by sex Ground reaction force measurements were shown as mean (standard deviation) F/M - force/body mass; RFD/M - rate of force development/body mass Statistically significant differences between sex (**p<0.01)

Measurments	Male	Female	p-value
F/M (kgf·kg^−1^)	1.46 (0.11)	1.34 (0.09)	**<0.001
RFD/M (kgf/s·kg^−1^)	11.81 (1.88)	10.76 (1.55)	**<0.001
Stable time (s)	1.06 (0.13)	1.03 (0.87)	**<0.001

**Table 3:** Ground reaction force measurements by age group Ground reaction force measurements were shown as mean (standard deviation) F/M - force/body mass; RFD/M - rate of force development/body mass Statistically significant differences between each age group by analysis of variance (**P<0.01)

	20–39	40–49	50–59	60–69	≥70	p-value
F/M (kgf·kg^−1^)						
Male	1.52 (0.12)	1.48 (0.12)	1.47 (0.10)	1.45 (0.10)	1.42 (0.12)	<0.001**
Female	1.36 (0.09)	1.36 (0.08)	1.34 (0.09)	1.33 (0.10)	1.29 (0.09)	<0.001**
RFD/M (kgf/s·kg^−1^)						
Male	12.64 (1.99)	12.08 (2.01)	11.90 (1.74)	11.47 (1.71)	11.18 (2.16)	<0.001**
Female	11.15 (1.65)	11.06 (1.43)	10.73 (1.47)	10.72 (1.61)	10.76 (1.55)	0.003**
Stable time (s)						
Male	1.05 (0.09)	1.08 (0.21)	1.05 (0.08)	1.06 (0.10)	1.08 (0.11)	0.370
Female	1.04 (0.10)	1.02 (0.08)	1.03 (0.08)	1.03 (0.09)	1.02 (0.10)	0.431

Table 4 illustrates the association between the GRF measurements and cardiovascular disease biomarkers. In terms of the F/M ratio, the multiple regression analysis results showed significant differences between males and females in terms of SBP, HDL-C, and PG. F/M was associated with SBP (β=-12.41, p=0.016 for male participants; β=-15.66, p=0.035 for female participants), HDL-C (β=22.59, p<0.001 for male participants; β=20.35, p=0.011 for female participants), and PG (β=-16.25, p=0.008 for male participants; β=-18.78, p=0.028 for female participants) in both male and female participants, and with TG levels in female participants (β=-55.12, p=0.025).

**Table 4:** Linear regression analysis for cardiovascular disease biomarkers and ground reaction force measurements adjusted for age HDL - high-density lipoprotein; LDL - low-density lipoprotein; HbA1c - hemoglobinA1c; F/M - force/body mass; RFD/M - rate of force development/body mass; CI - confidence interval *p<0.05, **p<0.01

	Male		Female	
	β (95%CI)	p-value	β (95%CI)	p-value
F/M				
Systolic blood pressure (mmHg)	−12.41 (−22.49– (−2.34))	0.016*	−15.66 (−30.22– (−1.11))	0.035*
Diastolic blood pressure (mmHg)	−3.04 (−10.95–4.87)	0.451	−8.59 (−20.31– 3.14)	0.151
HDL cholesterol (mg/dL)	22.59 (12.26–32.93)	<0.001**	20.35 (4.65–36.05)	0.011*
LDL cholesterol (mg/dL)	−5.06 (−25.07–14.96)	0.62	−9.99 (−40.69–20.71)	0.523
Triglycerides (mg/dL)	−23.23 (−95.66–49.21)	0.529	−55.12 (−103.15– (−7.08))	0.025*
Plasma glucose (mg/dL)	−16.25 (-28.28– (−4.22))	0.008**	−18.78 (−35.48– (−2.07))	0.028*
HbA1c (mmol/mol)	−0.35 (−0.76–0.06)	0.096	−0.50 (−1.01–0.02)	0.057
RFD/M				
Systolic blood pressure (mmHg)	−0.19 (−0.76–0.39)	0.53	−0.52 (−1.36–0.32)	0.226
Diastolic blood pressure (mmHg)	0.07 (−0.39–0.52)	0.772	−0.35 (−1.03–0.33)	0.311
HDL cholesterol (mg/dL)	0.57 (−0.03–1.17)	0.061	1.23 (0.321–2.13)	0.008**
LDL cholesterol (mg/dL)	−0.31 (−1.46–0.83)	0.59	−0.47 (−2.25–1.30)	0.599
Triglycerides (mg/dL)	0.06 (−4.09–4.20)	0.979	−4.31 (−7.07– (−1.55))	0.002**
Plasma glucose (mg/dL)	−0.63 (−1.32–0.06)	0.072	−0.93 (−1.90–0.03)	0.059
HbA1c (mmol/mol)	−0.02 (−0.04–0.01)	0.189	−0.03 (−0.06–0.00)	0.074
Stable time				
Systolic blood pressure (mmHg)	−0.75 (−8.93–7.44)	0.858	6.56 (−8.30–21.41)	0.386
Diastolic blood pressure (mmHg)	−6.03 (−12.42–0.36)	0.064	3.28 (−8.67–15.22)	0.539
HDL cholesterol (mg/dL)	−6.06 (−14.52–2.40)	0.16	−4.80 (−20.87–11.26)	0.557
LDL cholesterol (mg/dL)	7.43 (−8.76–23.62)	0.368	6.42 (−24.79–37.64)	0.686
Triglycerides (mg/dL)	−0.11 (−58.75–58.51)	0.997	42.54 (−6.40–91.48)	0.088
Plasma glucose (mg/dL)	15.57 (5.85–25.28)	0.002**	14.58 (−2.44–1.60)	0.093
HbA1c (mmol/mol)	0.55 (0.22–0.89)	0.001**	0.56 (0.037–1.075)	0.036*

Among female participants, significant differences were observed in TG levels. In the RFD group, significant differences were observed in both HDL-C and TG levels in the female participants. RFD/M was associated with HDL-C and TG levels (HDL-C: β=1.23, p=0.008; TG: β=-4.31, p=0.002).

Regarding stable time, significant differences in HbA1c levels were observed between males and females. Male participants showed a significant difference in PG. Stable time was associated with HbA1c in both male and female participants (β=0.55, p=0.001 for male participants; β=0.56, p=0.036 for female participants) and with PG in male participants (β=15.57, p=0.002).

Table 5 shows the association between GRF measurements and cardiovascular disease risk factors. The median (IQRs) of the three quartiles is shown in the table. Logistic regression analysis revealed that the adjusted odds ratios (aOR) for HT, HL, and DM were higher in Q1 than in Q3 for F/M. Logistic regression analysis showed that a lower F/M ratio was associated with HT, HL, and DM (HT: aOR=1.60, p=0.01; HL: aOR=1.75, p=0.001; DM: aOR=2.23, p=0.002).

**Table 5:** Logistic regression analysis for cardiovascular disease risk factor and tertile of ground reaction force measurements adjusted for age and sex † Tertile ground reaction force measurements were shown as medians (interquartile ranges). F/M - force/body mass; RFD/M - rate of force development/body mass; CI - confidence interval; aOR - adjusted odds ratio *p<0.05, **p<0.01

		Q3	Q2	Q1
F/M		1.54 (1.49–1.58) †	1.41 (1.38–1.44) †	1.30 (1.26–1.33) †
Hypertension	aOR (95%CI)	reference	1.09 (0.80–1.49)	1.60 (1.12–2.30) *
Hyperlipidemia	aOR (95%CI)	reference	1.39 (1.03–1.87) *	1.75 (1.24–2.46) **
Diabetes mellitus	aOR (95%CI)	reference	1.21 (0.75–1.95)	2.23 (1.33–3.75) **
RFD/M		13.20 (12.61–14.00) †	11.35 (10.98–11.73) †	9.65 (8.93–10.12) †
Hypertension	aOR (95%CI)	Reference	1.20 (0.88–1.63)	1.30 (0.94–1.78)
Hyperlipidemia	aOR (95%CI)	Reference	1.38 (1.03–1.85) *	1.46 (1.08–1.97) *
Diabetes mellitus	aOR (95%CI)	Reference	1.27 (0.79–2.07)	1.63 (1.01–2.62) *
Stable time		1.13 (1.09–1.19) †	1.04 (1.03–1.05) †	0.98 (0.95–0.99) †
Hypertension	aOR (95%CI)	reference	0.91 (0.67–1.23)	0.86 (0.64–1.15)
Hyperlipidemia	aOR (95%CI)	reference	0.98 (0.74–1.31)	0.79 (0.60–1.05)
Diabetes mellitus	aOR (95%CI)	reference	0.77 (0.51–1.18)	0.39 (0.24–0.64) **

In terms of HL, the ORs were higher for Q2 and Q1 than for Q3. For RFD/M, the ORs were higher in Q2 and Q1 than in Q3. For DM, the aOR was higher for Q1 than for Q3. A lower RFD/M ratio was associated with HL and DM (HL: aOR=1.46, p=0.013; DM: aOR=1.63, p=0.045).

Regarding the stable time, the aOR was lower in Q1 than in Q3 for DM. A shorter stable time was associated with DM (aOR=0.39, p<0.001).

## Discussion

This study assessed the association between the measured GRF during rising motion and cardiovascular disease biomarkers/risk factors. Our findings revealed that a decline in physical function was associated with increased risk factors across GRF values. Moreover, the specific risk factors associated with the different measurements varied depending on the magnitude of the GRF.

F/M was associated with SBP, HDL-C, and PG in both males and females in relation to cardiovascular disease biomarkers and with TG in females alone. Lawman et al. reported associations between relative grip strength and biomarkers and found associations between SBP and HDL-C, TG, PG, and plasma insulin levels in both males and females [7]. Lee et al. also reported an association between relative grip strength and these biomarkers [8]. F/M is reportedly associated with sarcopenia and knee extension muscle strength [1, 17]. There are reports on the relationship between grip strength and knee extension muscle strength [18]. Therefore, F/M can be considered as a parameter that indicates muscle mass or strength, and we believe that decreases in muscle strength and mass are related to cardiovascular disease biomarkers. Additionally, muscle may play a central role in protein metabolism [19], and we believe that this study demonstrates one aspect of this role.

RFD/M was found to be associated with HDL-C and TG levels in the females in our study. The results differed between the RFD/M and F/M conditions. This may be because RFD/M, unlike F/M, is a value significantly influenced by neurological factors [20]. The difference between male and female participants may be due to the hormonal effects in females. It has been reported that postmenopausal females tend to have decreased HDL-C and increased LDL-C and TG levels [21]. It has also been reported that muscle weakness and decreased skeletal muscle mass are observed in females after menopause [22]. Therefore, there may be a positive relationship between RFD/M and HDL-C and a negative relationship between RFD/M and TG in females. However, this notion merits further investigation. Stable time was associated with PG and HbA1c levels, and it has previously been reported to be associated with knee extensor strength [6]. It is also considered a phase during which stabilization of the standing posture is required after a rising motion [23]. Therefore, we believe that stable time is affected by muscle weakness and the ability to control posture, and we found a negative relationship between PG and HbA1c.

We then demonstrated the relationship between the GRF values and cardiovascular disease risk factors. Among the other risk factors, a low F/M ratio was associated with hypertension, hyperlipidemia, and diabetes mellitus. F/M has been reported to be associated with lower limb muscle strength and sarcopenia [1, 17]. Relative grip strength is a predictor of hypertension, hyperlipidemia, and diabetes mellitus [24-26]. Sarcopenia is reportedly associated with hypertension [27]. We believe that the correlation identified between the F/M ratio and cardiovascular disease risk factors supports these findings.

In the RFD/M group, lower values were associated with hyperlipidemia and diabetes mellitus. This was consistent with the results previously described by Monma et al. [25]. Vertical jumping requires instantaneous force and is associated with RFD [28]. The RFD/M in this study indicated that the RFD/M during the standing-up motion was related to hyperlipidemia and diabetes mellitus.

In terms of stable time, the rate of diabetes mellitus was lower in patients with shorter times. Monma et al. reported that one-leg balance was a predictor of diabetes mellitus [25]. We found that the shorter stable time was also related to diabetes mellitus, supporting this notion. However, decreased F/W and RFD/W ratios were also related to diabetes mellitus, and the possibility that these functional declines may represent confounding factors may not be ruled out. Although some elements of balance function are related to muscle strength, many other factors are also considered independent predictors [29]. Therefore, a more detailed study on the relationship between ST and diabetes mellitus is warranted.

In this study, GRF measurements from the standing-up motion were associated with cardiovascular disease risk factors. It has been suggested that a decrease in lower limb function due to standing movements using a GRF measuring device may indicate an increase in cardiovascular disease risk factors.

The current study aimed to evaluate GRF measurements as a crucial parameter within a healthy adult population to identify and mitigate potential future risk factors. One notable strength of this study lies in its inclusion of participants spanning a wide age range; additionally, the assessment was done through a readily applicable sit-to-stand movement. A limitation of this study is that it was designed to evaluate only the inhabitants of a single geographical area, and all participants were already undergoing health check-ups. Further research is warranted to explore the longitudinal implications of impaired lower limb function in the context of cardiovascular disease development and to ascertain the predictive value of this assessment modality in larger, diverse populations.

## Conclusions

Our results suggest that a decrease in lower limb function due to sit-to-stand movement using a GRF measuring device may indicate an increase in the risk factors for cardiovascular disease. These findings underscore the potential clinical utility of incorporating sit-to-stand movement assessments utilizing GRF measurement devices as a supplementary tool in cardiovascular risk assessment protocols.

## Appendices

**Table 6:** Lifestyle questionnaire

Questions		Answer choices
1	Are you currently using an anti-hypertensive drug?	Yes/No
2	Are you currently using insulin injection or an antidiabetic (hypoglycemic) drug?	Yes/ No
3	Are you currently using an anti-cholesteric agent?	Yes/ No
4	Have you ever been diagnosed as having a stroke (cerebral hemorrhage or infarction)	Yes/ No
5	Have you ever been diagnosed as having heart disease (angina pectoris or myocardial infarction) by a physician or had medical treatment?	Yes/ No
6	Have you ever been diagnosed as having chronic renal failure by a physician or underwent artificial dialysis?	Yes/ No
7	Have you ever been diagnosed with anemia?	Yes/ No
8	Have you smoked in the last month?	Yes/ No
9	Have you put on weight by 10 kg since your 20s?	Yes/ No
10	Have you exercised more than 30 minutes per session, more than two times per week, for more than 1 year?	Yes/ No
11	Do you walk daily or perform other physical activities equal to walking for more than 1 hour daily?	Yes/ No
12	Do you walk faster than those in the same age group as you?	Yes/ No
13	Which of the following applies when chewing meals?	Chew well/ Sometimes difficult/ Always difficult
14	Do you eat faster than others?	Fast/Normal/Slow
15	Do you have dinner within 2 hours before going to bed more than three times a week?	Yes/ No
16	Do you take snacks or sweet drinks in addition to the three meals regularly?	Everyday/Sometimes/ Rarely
17	Do you skip breakfast more than three times a week?	Yes/No
18	How often do you drink alcohol (such as sake, shochu, beer, whisky etc.)?	Everyday/Sometimes/ None
19	The day you drink, how much alcohol do you consume?	Less than 180 ml/ 180–360 ml/ 360–540 ml/ More than 540 ml
20	Do you sleep enough?	Yes/No
